# An Essay on Sore Throat, or Chronic Pharyngitis

**Published:** 1853-08

**Authors:** 


*An Essay on Sore Throat, or Chronic Pharyngitis.* Read before the Memphis Medical Society, May, 1852. By A. P. MERRILL, M. D.

This disease is of frequent occurrence, sometimes being caused by acute inflammation of the lining membranes of the throat, and sometimes by scarlatina, measles, exposure to cold, etc. It consists of a subacute inflammation or hyperæmic condition of the mucous membrane of the pharynx, frequently involving the tonsils and palate, the glottis and epiglottis, and extending into the nares and Eustachian tubes, and even into the external meatus of the ear. The membrane presents in these cases a highly vascular condition, and sometimes an evident thickening or hypertrophy, and not unfrequently an œdematous tendency.

In cases of severity there is a pretty constant tickling in the throat, producing a disposition to cough or to hack, a feeling of irritation and roughness about the palate and glottis, painful deglutition, and an increase of all the unpleasant symptoms consequent upon an overstraining of the voice, followed by hoarseness, and in some instances by temporary or permanent aphonia. In such cases, the secretions from this membrane are nearly suspended; but with a partial abatement of vascular action, a viscid mucus is formed, which adheres closely to the surface, and by the irritation it produces, causes coughing.

As in other cases of subacute inflammation of the mucous membranes, a long continuance of this disease sometimes leads to ulcerations. The ulcers are generally of a phagedenic character, spreading rapidly, with considerable waste of substance, and exceedingly sensitive to the touch. The act of deglutition, while these ulcers exist, is attended by acute pain, like the thrust of sharp instruments into the flesh. Sometimes the ulcerative process is of the pustular character, producing pustules or pimples considerably elevated, and filled with matter. This form of the disease has been called "Follicular Pharyngitis."

Chronic Pharyngitis sometimes has its beginning in early youth, and continues without any very urgent symptoms for many years. Persons troubled with this form of the disease are apt to experience a tickling in the throat, attended by a collection of viscid mucus, and a disposition to cough, or to hawk, upon every sudden transition from warm to cold air, and particularly upon first going out in the morning, in cold and damp weather. But these symptoms pass off as the day advances and the system becomes warmed by exercise.

When the hyperæmia and turgescence are much increased from any cause, a rupture of some of the engorged blood-vessels is a common occurrence, and the patient ejects mucus tinged with blood, which is hawked up from the throat. By close attention to this act, it is easy to distinguish such cases from those of bronchitis, in which bloody sputum is coughed up from the lungs. And it is not difficult for an experienced observer to distinguish pharyngitis in all its stages from laryngitis, trachitis and bronchitis, even by the act of coughing or of ejecting sputum from the throat by convulsive efforts, which are short of coughing, generally called hawking or clearing of the throat. Pharyngitis leads to the establishment of those other diseases, however, by the extension of the diseased action into the air passages, and then the two affections exist together. This is the result so much to be dreaded, and which makes the early and skillful treatment of the disease of the utmost importance.

Habitual coughing, to a greater or less extent, is a common symptom attending pharyngitis, and it is one which requires our particular attention. I believe it is a well ascertained fact, that the lungs cannot be exercised by frequent and long continued coughing, from any cause, without danger of the most serious consequences. The bronchial tubes are certain, sooner or later, to take on an excited and diseased condition from this convulsive movement, with every danger of hemorrhage and ulceration supervening, attended by hectic fever and other characteristic symptoms of phthisis pulmonalis. Indeed a large majority of the cases of consumption which I have met with, have had their origin in a cough proceeding from the irritation caused by chronic pharyngitis. Persons of a strumous habit, and those who labor under the disadvantages of a hereditary taint, are perhaps more liable to this result than others, and experience its fatal influence in a shorter period of time; but the exciting cause is the same, and the course of the disease very similar.

This disease is commonly called *bronchitis,* but this is an erroneous designation, for the bronchial tubes are not primarily affected, and when they do become seriously involved in the diseased action, it is not long before a name of more fearful import is assigned to it. We hear then of consumption and not bronchitis.

Public speaking is one of the most exciting causes of pharyngitis, and clergymen, therefore, appear to be particularly obnoxious to it. This arises mainly from the unnatural and labored efforts which they are accustomed to make, together with their want of knowledge of the physiology and philosophy of the human voice. These efforts are in some cases repeated very often, in consequence of the high estimate which Christians of the present day place upon much preaching; and they are frequently made in over-heated and corrupted atmosphere, the transition from which to a cooler and purer medium, in a state of fatigue and exhaustion, has a strong tendency to enhance the ill effects.

The usual remedies for this disease are counter-irritants externally, and stimulating and escharotic applications to the diseased surface. The former consist of blisters and various kinds of stimulating embrocations, accompanied by the use of flannel or silk to protect the throat from the influence of the cold air. The beard is sometimes permitted to grow for the same purpose. Sometimes a contrary course has been pursued, and the whole neck has been bared to the weather, with frequent cold ablutions, general cold bathing, and even the application of ice. These various remedies have their advocates, and no doubt all have proved useful under certain conditions.

The applications made to the throat, internally, are, capsicum, the sulphates of copper and zinc, nitrate of silver, etc. Capsicum has been found very useful in many cases, from its actively stimulating effects upon the torpid vessels, by which they are made to contract their calibres, and thus reduce the prevailing hyperæmia. A healthy secretion of mucus sometimes follows this application, and a consequent relief of urgent symptoms. The principal fault in its application consists in its being prescribed as a gargle. Used as such, in the general acceptation of the term, it does not reach, to much extent, the diseased surface, but comes in contact principally with the mouth and lips, annoying the patient by its burning stimulation, without much influence over the disease. The best way of applying it is, by the use of a camel's hair brush, or a bit of sponge, using a strong decoction, and washing it well over the diseased surface. Or, if the patient throws back his head, and by the use of a small spoon carries a few drops far back upon the root of the tongue, and then swallows with the throat in this straightened position, he will bring the liquid into contact with a considerable portion of the diseased membrane, and avoid at the same time, its unpleasant effects upon the lips.

The nitrate of silver has latterly been in higher repute in the treatment of this disease, than any other topical remedy; and it is at this time, I believe, more generally prescribed. It is no doubt valuable, and I have myself witnessed the happiest effects from its application, both in solution and in substance. In one case of considerable violence, however, phagedenic ulcers formed in the throat while the patient was applying a strong solution of the nitrate of silver to the part affected, three times a day. These ulcers were cured, and the disease greatly relieved by the substitution of a solution of sulphate of copper. But I need not pursue this subject of treatment, which is so familiar to you all, and will therefore proceed, without further remark, to the special object of this paper.

It will be recollected by some of the members present, that several months ago I invited the attention of the society, verbally, to the use of iodine as a topical application to the internal throat, in this disease. I had at that time only a limited experience with the remedy, but had witnessed remarkable effects from its use in several cases. And now, after some further trials by myself and others, I am so far confirmed in my favorable opinion of the remedy, as to feel myself called upon by a sense of duty to the profession, to the society, and to mankind, to direct your attention to the subject in a more formal manner.

Having been in the constant use of this remedy in my practice for more than a year, and having succeeded in relieving several cases of chronic pharyngitis with it, which had resisted the use of other active means of cure, I now venture to recommend it as a valuable remedy, and to request the members of this society to put its merits to the test of experiment, as they have opportunity, and to report the result to the society.

The following is a convenient formula, which may be applied to the throat, with a camel's hair brush, once or twice a day, or as often as the patient feels a tickling sensation and a desire to cough. Persons afflicted with this disease will find it advantageous to have the remedy at hand during the night, so that the cough may be arrested at once, whenever it becomes troublesome:

| | | |
|---|---|---|
| ℞ | Iodide of potassium, . . | ʒj. |
| | Iodine, . . . . . | ʒss. |
| | Water, . . . . . . | ℥j. |
| | Gum Arabic, . . . . | ʒij. |
| | White sugar, . . . . | ʒij.—Mix. |

For the external meatus of the ear the following is used:

| | | |
|---|---|---|
| ℞ | Iodide of potassium, . . | gr. vj. |
| | Iodine, . . . . . | gr. iij. |
| | Water, . . . . . . | ℥j. |
| | Glycerine, . . . . . | ʒij.—Mix. |

In some cases of long standing, this solution proves to be too weak. It should be of a strength to produce a slightly burning sensation. The glycerine is important on account of the dryness of the meatus, which nearly always attends these cases, and this may be increased at pleasure. A few drops may be put in the ear once or twice a day, and in cases of severity, or insensibility, a lock of cotton may be charged with the solution and suffered to remain in the ear. It must be borne in mind, however, that in many cases of diseased ear, the meatus externus is extremely sensitive, and will not bear the application of iodine. Proper caution should therefore be observed in its use, lest it prove too stimulating and give pain.—*N. Orleans Journal.*

